# Logic model development through a feasibility RCT for a group-based weight management programme

**DOI:** 10.1136/bmjopen-2024-094569

**Published:** 2026-03-24

**Authors:** Rod Sheaff, Shokraneh Moghadam, Laura Hollands, Lily Hawkins, Dawn Swancutt, Jenny Lloyd, Jonathan Pinkney, Mark Tarrant

**Affiliations:** 1 Peninsula Medical School, Plymouth University, Plymouth, UK; 2 University of Bristol, Bristol, UK; 3 School of Psychology, Plymouth University, Plymouth, UK; 4 Occupational Health and Wellbeing Service, West Glasgow ACH, Glasgow, UK; 5 Faculty of Medicine & Dentistry, University of Plymouth, Plymouth, UK; 6 Department of Health and Community Sciences, University of Exeter, Exeter, UK

**Keywords:** Obesity, Behavior, Psychosocial Intervention, Weight Gain, Organisation of health services

## Abstract

**Objectives:**

Clinical psychology interventions for reducing obesity have developed alongside pharmacological and surgical treatments, but usually as interventions for individual patients. Any healthcare intervention rests on a logic model: assumptions that through specific physical and social mechanisms, it will produce certain intended outcomes, provided that conducive background conditions (‘contexts’) exist. Using evidence from the feasibility trial preceding a full randomised controlled trial (RCT), this paper assesses the empirical validity of the initial logic model of a new group-based weight management intervention: PROGROUP, designed for patients with body mass index (BMI) ≥40 kg/m^2^ or ≥35 kg/m^2^ with comorbidities. We aimed to test whether:

1. PROGROUP’s programme components produce the intended outcomes at all, whatever their size and how.
2. The intervention can practicably be implemented (‘delivered’) as designed.
3. How the programme and delivery components affected each other.

**Design:**

Multimethod proof-of-concept study by means of realist evaluation of the initial PROGROUP logic model. We:

1. Elicited the logic model underlying the intervention design.
2. Compared these assumptions with data from a pre-RCT feasibility study in two English and one Welsh National Health Service (NHS) sites during 2021–2023.
3. Revised the logic model in light of the data, noting how much variation in delivery the programme components (therapeutic mechanisms) could tolerate.

**Setting:**

Specialised ambulatory mental health services in the English NHS.

**Participants:**

Adults with severe obesity (BMI ≥40 kg/m^2^ or ≥35 kg/m^2^ with comorbidities).

**Intervention:**

Group-building techniques to enhance group members’ adoption of evidence-based methods of behaviour change affecting their dietary behaviour and physical activity.

**Primary and secondary outcome measures:**

Qualitative outcomes. What kinds of:

1. Mechanisms were established and triggered by the attempt to implement PROGROUP (secondary outcome, see objective 1 above).
2. Patient behaviours resulted and whether patients sustained them after the intervention ended (primary outcome; see objective 1 above); and what kinds of context affected that (objectives 2 and 3).

Quantitative measures not used.

**Results:**

The initial logic model assumed that the following sequence of mechanisms would produce weight loss: referral from GP to specialist weight management services; further referral to PROGROUP; preparatory individual consultation; facilitated group sessions produce a group identity; group identity reinforces weight management capability and motivation; further individual consultations adjust for individual circumstances; behaviour change outside the treatment setting, producing weight loss. Contexts necessary for these mechanisms to work included: sufficient catchment population; group size, continuity and membership retention; suitable location; facilitator training; and practical support outside the treatment setting.

**Conclusions:**

The findings suggested revisions to the logic model, but more in the delivery components and contextual assumptions than the core therapeutic mechanisms. There was scope to simplify the referral mechanisms. Different professions could implement the model. A realist evaluation of a pre-RCT feasibility study can be used to make the intervention’s logic model more securely evidence-based, serving as a proof-of-concept test for the intervention. It indicated the conditions under which such group psychological interventions might be more widely used.

**Trial registration number:**

ISRCTN22088800.

**STRENGTHS AND LIMITATIONS OF THIS STUDY**

- Logic model development is a method for empirically testing and developing the design of an intervention before full-scale clinical trial or widespread adoption, to make it more securely evidence-based, discover which contexts aid its implementation, and how much variation in its implementation the intervention can tolerate.
- The method is especially relevant to feasibility trials.
- It was designed to analyse qualitatively what kinds of causal connection(s) obtained between the intervention mechanisms and their outcomes, and which types of context potentiated or weakened those mechanisms.
- The method did not quantify the relationships between mechanisms, contexts and outcomes.

Psychological interventions for reducing obesity have until recently usually been interventions for individual patients. Long-established group interventions for groups have generally addressed class 1 obesity, but in classes 2 and 3, the psychological issues and comorbidity are more complex and group interventions are less well explored. This paper therefore assesses the empirical validity of the initial logic model of a new group-based weight management intervention, PROGROUP, and whether it appears extensible for use with other groups. Any healthcare intervention rests on a logic model^1^: assumptions that the intervention will trigger specific physical and social mechanisms which produce certain intended outcomes, provided that conducive background conditions (‘contexts’) obtain.^2^ Implementing the intervention puts these assumptions to a practical, empirical test. In case the assumptions are false, or only true under certain conditions, it is prudent to test them on a small scale to obtain prima facie evidence as to whether, or when, they apply. Using that evidence, the initial logic model can then be strengthened before risking a larger implementation. This paper reports, therefore, how evidence from the feasibility trial preceding a full randomised controlled trial (RCT) was used to develop the PROGROUP logic model.

## Background

Obesity has been becoming more prevalent world-wide for over 40 years.^3^ It increases the risks of cancer, cardiovascular disease, diabetes, kidney diseases, maternal and neo-natal health problems, and musculoskeletal disorders.^4^ Severe obesity reduces life expectancy by 8–10 years and self-reported quality of life.^5 6^ It directly exacerbates depression and other mental health problems,^5 7^ but also exacerbates them indirectly through weight stigma.^8^ Treatments have included bariatric surgery (various procedures,^9^ but increasingly laparoscopic). Among the pharmacotherapies,^10^ semaglutide is an important new medication,^11^ effective both alone and when combined with lifestyle interventions.^12 13^ Even without semaglutide, these lifestyle interventions include exercise programmes,^14 15^ low-fat or low-carbohydrate diets,^16 17^ intensive dietary strategies^18^ and individual counselling, whether using motivational interviews,^19^ patient education,^20^ psychotherapy,^21^ cognitive-behavioural therapy (CBT) or behavioural theory. Behavioural therapy appears more effective than CBT for managing binge eating, and the reverse for non-binge eating. For obesity combined with depression, they do not significantly differ.^22^ To varying degrees, they increase patient adherence to dietary and exercise advice.^23 24^ Weight loss after 5 years was associated with the initial weight loss that group interventions produced, and with ongoing access to the programme after research into the intervention ended.^25^ However, little of this evidence concerns severe obesity specifically.

These interventions usually treat patients individually. However, it appears that group-based interventions may also enhance patients’ motivation to follow advice about diet and exercise, and their capability for doing so.^26^ How far that occurs depends on how the group itself works internally,^27^ becoming more likely insofar as the members identify with the group as a whole.^28 29^

A new intervention, ‘PROGROUP’, therefore aimed at developing the social identity of patients within treatment groups so as to enhance their motivation to, and capability in, following advice about diet and exercise. Targeted on people with severe obesity (defined as body mass index (BMI) ≥40 kg/m^2^ or ≥35 kg/m^2^ with comorbidities),^30^ it adopted group-building techniques to enhance group members’ adoption of evidence-based methods of behaviour change^31 32^ and so shape the psychological mechanisms underpinning their dietary behaviour and physical activity including regulatory processes, habits and broad lifestyle values. These behavioural changes were intended to produce a sustained reduction in BMI. PROGROUP was not just theory-congruent^33 34^ but theory-based, applying assumptions from social identity theory,^35^ self-categorisation theory^36^ and the social identity approach to health more generally.^37^ The social identity approach predicts that developing a sense of social identification with each other during group sessions^29^ enhances group members’ ability to engage with the programme’s behaviour change content and make the corresponding lifestyle changes. From ongoing access to the group and its collective resources, its members are expected to derive a sense of social support and feel less isolated when confronting weight management challenges in their everyday lives, which would reinforce their capability and motivation to attempt the behavioural changes that weight management involves.^29 38 39^

A logic model includes programme components, the mechanisms which produce its intended outcomes; and delivery components, the organisational activities and resources through which the programme components are implemented.^33^ In group interventions especially, the two sets of components are intercalated, raising the question of how each affects the other. Outside healthcare, such studies are numerous although diffuse, but in healthcare, they are sparse.

Before a full-scale RCT, it would seem prudent to test the assumptions on which the intervention rests. The objectives of this study were therefore to examine:

1. whether PROGROUP’s programme components produce the intended outcomes at all, whatever their size, and how. whether the intervention can practicably be implemented as designed.
2. how the programme and delivery components of PROGROUP affected each other, in particular how much, and what kinds, or variation in delivery components a group intervention such as PROGROUP can tolerate without compromising the programme elements.

Objectives 1 and 2 constitute a qualitative proof-of-concept test of the initial logic model of the intervention as a whole, rather than testing its component behaviour change techniques. Insofar as a qualitative analysis can be said to have primary and secondary outcomes, objective (1) above is the primary outcome. This paper uses evidence from a feasibility trial^40^ of the PROGROUP model to address these objectives.

## Methods

Realist evaluation is the method of first making explicit, and then empirically testing, the aforementioned assumptions about the physical and social mechanisms that the intervention applies, what outcomes these mechanisms produce, and in what contexts. The method equally accommodates natural and social causal processes.^41^ PROGROUP used both. First, therefore, we formulated in a realist way the initial logic model which PROGROUP embodied, including the theoretical assumptions on which its main mechanisms (programme elements) were based. The second stage was to use a feasibility study as a proof-of-concept test of that logic model, which took place in one Welsh and two English NHS sites during 2021–2023. While the researchers trained the PROGROUP facilitators, it was these NHS staff who delivered PROGROUP to patients.Table 1 lists the data sources.

**Table 1:** Data sources

Source	N	Contribution to findings
Patient interviews	21	First-hand participant and service provider accounts
Facilitator and manager interviews	13	
Researcher field notes and observation of group sessions.	10	Observation of mechanisms and contexts.
Facilitator on-line feedback and support sessions (weekly)	20	First-hand participant and service provider accounts
Post-feasibility study stakeholder meetings (including past patients, managers, facilitators)	2	
Patient end-of-programme questionnaire	94	4 open questions elicited participant accounts
Systematic review: existing published and peer-reviewed ‘grey’ studies.	90	Secondary data
RCT set-up: participating sites	60	Combined number of email messages, site visits and set-up discussions of successive phases of the study: facilitators and barriers
RCT set-up: non-participating sites	18	Combined number of email messages, site visits and set-up discussions of successive phases of the study: facilitators and barriers

RCT, randomised controlled trial.

Third, we systematically compared these data with the initial logic model, revising the model when the two conflicted (see ‘Logic Model Revision’ below). Differences between the sites which could and could not implement PROGROUP revealed delivery barriers and facilitators. Differences in the ways that different sites implemented PROGROUP showed how much variation in its delivery components the programme components could tolerate (ie, how much the implementation of PROGROUP could vary, without compromising its outcomes). By analysing what delivery conditions were necessary for each mechanism, it also became possible to hypothesise what further, as yet unobserved, variations in delivery components that programme component might also tolerate. For example, data on where the intervention was given (see below) suggested which characteristics differentiated practicable from impracticable locations in which to deliver PROGROUP, and the range of practicable venues.

A risk of self-justification bias arises when the researchers who devised an intervention implement it and report the outcomes. To prevent retrospective adjustment of the aims and research questions as findings emerged, before starting, we published the feasibility trial protocol^40^ and, in the original grant application, the PROGROUP programme components. Data were analysed by researchers (RS, SM, JL and LHo) who had not been involved in designing the intervention, but collaborated with the designers in evaluating it. As a falsificationist precaution,^42^ we also looked for evidence against the logic model. In reporting the findings, we comply with Standards for QUality Improvement Reporting Excellence (SQUIRE) 2.0 guidelines.^43^

### Patient and public involvement

During the study design and subsequently, a Patient Advisory Group and a Steering Committee with independent patient representation provided patient and public involvement (PPI). One of the co-applicants for study funding was a PPI representative with personal experience of weight management services and advocacy, and who has been directly involved in the study ever since. PPI representatives advised on study design and protocol development, all patient-facing written material, aspects of data collection including topic guides, and designing the full RCT. When that is completed, they will contribute to disseminating the results, including those reported below.

### Initial logic model

**Figure 1:**
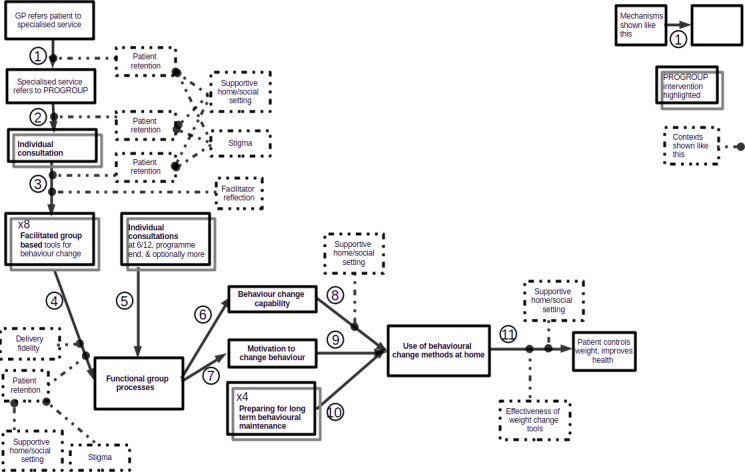
PROGROUP initial logic model represents the initial PROGROUP logic model. GP, general practitioner.

Figure 1 shows the initial PROGROUP logic model including the contexts initially assumed to moderate its effects.

Its main programme components, that is, those expected to produce the intended outcomes, were mechanisms 3, 4, 5, 6, 7, 8, 9, 10 and 11. In light of an initial individual assessment, a facilitator would adjust (mechanism 3) and supplement (mechanism 5) the contents of the facilitated group sessions, which would produce (mechanism 4) positive group dynamics, including social connection, support and influence, and group norms. If group members had a favourable experience of the first few group meetings, these dynamics would become self-reinforcing. They would both equip (mechanism 6) and motivate (mechanism 7) group members to undertake personal weight maintenance activities advocated within the programme content, thereby changing the members’ health values and aligning their sense of ‘who I am’ with the identity and values evolved during the course of the intervention. These effects would extend beyond the group setting into the participants’ social and family settings (mechanisms 8, 9). In-group maintenance activities would reinforce these effects (mechanism 10) and support patients to modify their environments in ways that are conducive to the values developed within the programme. Behaviour change outside the group setting would finally result (mechanism 11) in patients more actively managing their behaviour with the health benefits suggested above. These outcomes would develop both during and after the intervention.

Only mechanism 1 was purely a delivery component, i.e. one that did not directly produce the intended outcomes but helped to implement the programme components that did. Mechanism 2 combined patient recruitment (delivery component) and screening referrals for suitability (programme component). Similarly, as mechanism 3 facilitators prepared patients for the first group session by discussing any anxiety about attending (programme component), followed by setting up group meetings (delivery component). Group behavioural maintenance activity had as its delivery component the coordination of group activity, and as its programme component further reinforcement of social group identity and behaviour change in other settings, so that the group would sustain these activities after the PROGROUP sessions end (mechanism 10).

PROGROUP’s initial logic model assumed that certain further conditions (‘contexts’, in realist terms) also affected the programme components. One was patient retention during mechanisms 1–4, which the logic model assumed would depend in turn on a positive early experience of the group sessions. Others were that weight stigma did not inhibit participation in PROGROUP, and environments outside the treatment setting (eg, friendship and peer networks, vocational networks, home and family) were supportive. One intervention activity was therefore to get patients to map these group connections outwith the treatment setting and consider their compatibility with the values espoused during the intervention. Fidelity in programme delivery was assumed to be an important context for Mechanisms 4 and 10. Supportive environments outside the treatment setting were also assumed to affect group members’ motivation and ability to apply elsewhere the methods that they learnt in the group (mechanisms 6–10).

### Feasibility

We present the findings stepwise following the initial logic model (figure 1), reporting how each mechanism worked or did not, and in what contexts. Whether the use of behavioural change methods continued outside the treatment setting after the 2 years of research reported here, and did indeed enable patients to control their weight in the longer term, and so improve their health (mechanism 11), was a question beyond the feasibility study remit. An RCT to evaluate it began in 2024.

#### General practitioner refers patient (mechanism)

The first mechanism was that general practitioners (GPs) selected patients who might benefit from specialist weight management services. Like the All-Party Parliamentary Group on Obesity,^44^ we found that communication between weight management service staff and other clinicians was at times slow or liable to interruption. Referral routes were sometimes complicated.

As contexts, mechanism 1 presupposed that local specialist weight management services existed at all, which they did not in four prospective RCT sites, nor some others.^44^ A Public Health England survey^45^ suggested that only 21% of Clinical Commissioning Groups had such a service in their area, but the low response rate suggests that this figure may underestimate service coverage. Barriers to commissioning such services included lack of evidence of what works and disjointed care pathways. For one of our study sites, the shortage of potential facilitators for the group was a further obstacle, exacerbated by group meeting locations and schedules (see below). A non-study site GP network was reportedly setting up specialist weight management services of their own, which raises the question of whether other providers besides a community health service, such as a GP consortium, hospital out-patients department (OPD) or (outside the UK) polyclinic could equally deliver PROGROUP.

The intended outcome was a flow of suitable patient referrals to specialist services. During the study period, the pandemic limited in-person appointments, increasing referral and waiting times and causing this patient group particular anxiety because of their heightened risk of poor health outcomes from COVID if they caught it. Although healthcare professionals’ negative attitudes to obesity have been reported elsewhere,^46^ our informants did not mention obesity stigma as a context influencing referrals.

#### Specialist service refers patient to PROGROUP (mechanism 2)

Next, weight management services staff screened the referrals and referred suitable patients for a first individual consultation with a PROGROUP facilitator. Across sites, the facilitators included public health practitioners (graduates but not necessarily professionally registered), dieticians and physiotherapists, whose common characteristic was the generic skill of being able to provide National Institute for Health and Care Excellence-compliant dietary or nutrition care. At one site, patients whose needs were obviously urgent or complex were referred from less specialised services and a multidisciplinary team did the screening. At another,

Sometimes there was signposting to other agencies, erm, and sometimes […] if the clinician felt that the, the service matched the patient’s needs we would sign them up. But again it was offering an opt in […] asking people always to keep reaffirming what they wanted (Staff 01).

Staff assessed whether patients had mental health or physical co-morbidities, and at one site their home circumstances. For instance:

if somebody’s gone through a significant bereavement then and actually, there’s no way they’re going to be making these sorts of [behavioural] changes […] we can say, ‘Well we can postpone this until this is appropriate for you’ (Staff 02).

Once screened as suitable for PROGROUP, the patient might still have a long wait for their first individual consultation. This one was referred

in March, so April, May [came], I went up to university and […] Then I was meant to have an appointment with her, I think that was about November, but then it was cancelled, and then she’d gone off sick or something, so I then had this chap calling me… that was in November (Patient 1008).

Although PROGROUP neither focuses on weight per se nor sets personal weight loss goals, it was normal practice in the study sites to record patients’ BMI during the month before they joined a weight management programme. However, general practice measurements of patients’ height and weight were often lacking or inaccurate, as others have found.^47^ Some patients were reluctant to disclose or ‘dreaded’ to know their own BMI, and even refused to be weighed. Conversely, patients valued staff being non-judgmental (about their weight):

to have someone listen to you not judging and actually being able to say this is how I feel and you know, this is how it’s affect[ed] me all my life (Patient 2057).

Weight stigma was thus an impeding context for Mechanism 2. Waiting times often reflected organisational contexts: staff turnover, staff shortages and, during 2021–2023, the re-structuring of NHS commissioning into ‘Integrated Care Systems’. The COVID pandemic and NHS capacity issues at the time made it difficult for some staff to commit time to train as facilitators. The size of the population served was another important context. Small sites required several months to recruit the necessary 12–18 patients, especially one rural site with a small catchment population. Access to PROGROUP for suitable patients was the intended outcome of mechanisms 1 and 2, but they gave patients in the larger sites swifter access to PROGROUP.

#### From individual consultation to patient group (mechanism 3)

A preliminary consultation to assess and discuss treatment needs, options and preferences was already common clinical practice. Patient interviewees described the initial consultation in such terms as:

that was on the telephone and the person that I spoke with was very nice and was interested took details and asked me questions. And I was able to speak to her freely, so that was very positive (Patient 2054).

Initial consultation by video-link or telephone emerged as alternative delivery options. Group sessions (see below) were always face-to-face.

An emergent selection criterion was thus whether the patient was willing to make the practical commitment to group activity that PROGROUP requires. An unforeseen selection question was whether a group could contain two people related to each other, potentially disrupting group functioning since the two might either be closer or (as in one case in the present study) more antagonistic than the other group members. However, the circumstances linking these two people were traumatic and unusual.

For facilitators, the intended outcomes of these consultations were to prepare patients for the first group meeting, enable it to be organised and adapt group activities correspondingly, but by adjusting or adding to, not subtracting from, the core content stipulated in the logic model. In practice, though, flexible delivery of the group sessions (see below) appeared to stem less from the individual consultation than from the facilitators responding to the emergent characteristics of the group when it met. Nevertheless, the individual consultations did help the facilitators understand patients’ needs, backgrounds and similarities that the group sessions could draw on. After the present study, the session notes were therefore adapted to show facilitators where they could deliver the session contents more flexibly.

Cost, time, distance and facilitator availability were contexts that prevented having the initial consultation at the patient’s home.

#### Facilitated group presentations establish functional group processes (mechanism 4)

As the initial logic model assumed, most patients recognised they had a common interest joining the group:

it was a good thing I did [join the group], because, obviously, we’re all the same sort of ilk in a way, problems with food and overweight, and it was quite good having a group of people with the same thing (Patient 2007).

The one exception was a patient awaiting bariatric surgery who saw little point in being on the programme.

A first reported outcome of this mechanism was, as intended, to stimulate mutual support among the group members by revealing their common experiences and interests. Thus,

shall we say two thirds of the way through, everyone had got over those bits of anxiety and shyness and [… were] involving themselves, discussing various things […] We’ve all been there, we all had the same issues, we'd all experienced that (Patient 3009).

group members were sometimes relieved to learn that their weight management problems were not unique. Having a non-judgmental facilitator and group removed feelings of self-blame which could otherwise serve as a barrier to motivation and empowerment and helped build patients’ shared understanding:

The way they [the group] talk about it was that they spend their whole life feeling like the odd one out […] and now they’re in a group of people where they’re like ‘oh they get me, I can talk freely about it, nobody judges me, nobody criticises me, nobody’s sitting there thinking ah you’re just lazy or greedy’ (Facilitator 06).

One facilitator asserted that despite there being enthusiastic, and for that reason dominant, members who talked a lot, planned pair work meant the less dominant patients could also talk. Indeed, group members supported each other at difficult moments:

when we were talking about stress [… one patient] became very very upset […] everybody in the group rallied around her and picked her back up (Facilitator 02).

As a context affecting mechanism 4, group connectedness increased as time passed. Group members’ increasing knowledge of each other also strengthened the emergent group processes and sense of connectedness. Groups had to be large enough to withstand drop-outs due to family member ill health, patients moving out of the area and transport difficulties, besides single sessions missed due to holidays, bereavements and accidents. In practice, attendance by five or more patients appeared necessary to maintain the group sessions’ motivational effects. Attending a three- and then a two-person ‘group’ was demotivating. Once,

it was just one [other patient] and me. […] when there’s two of us, it’s quite hard, because it’s like doing it one to one (Patient 1015).

negative comments about the intervention mostly concerned this problem. In response, the site offered on-line sessions but nobody accepted them and the group closed.

Location was another context affecting how, or whether, facilitation had its intended effects. Two sites had difficulty in finding a clinic space large enough for group work and accessible for people with limited mobility. Relocating from hospital to a community centre solved that problem, but then some patients, especially from rural areas, faced long journeys to group meetings. Transport problems, reflecting the geographical dispersal of a rural population, thus exacerbated group attrition. To help retain patients one site reimbursed patients’ parking costs, bus fares or, exceptionally (and unlike most other services), taxis, which also helped patients arrive punctually. The scheduling of group sessions also had to fit participants’ circumstances. One site arranged Saturday meetings because many patients worked during the week, but the facilitators wanted their weekends free and therefore wrote to patients’ employers asking them to grant patients week-day leave to attend.

Staffing pressures made the requirement of 4 or 5 days of continuous face-to-face training unappealing for potential new facilitators. The initial logic model assumed that newly-recruited PROGROUP facilitators would only have limited knowledge of the principles of diet and aspects of weight-related behaviour. In practice, though, the facilitators (and some patients) already had that knowledge, but not necessarily knowledge of managing group processes.

#### Individual consultations contribute to functional group processes (mechanism 5)

For patients, second and third individual consultations served to address practical weight management issues that the group sessions had not (eg, how weight affects snoring). The only contextual factor was that, to reduce staff workload, sites considered scheduling each round of individual appointments in a single clinic session, or as on-line consultations. Few participants mentioned the second and subsequent individual consultations unprompted, and those who did seemed to find them rather inconsequential:

they asked me to come […] to do a weigh-in and take my blood pressure, and that was it (Patient 2018).

although this patient also mentioned getting a hospital referral for breathing difficulties.

#### Group processes increase behaviour change capability (mechanism 6)

Group sessions helped patients develop and share skills. One group:

did a study [as part of the intervention, not the feasibility study] […] about a lady who always went home and did XYZ, but if you changed XYZ, would the outcome be different? […] that made me think more about… like this morning, I woke up thinking. ‘Right, where can I buy something nice? I’ll have some chocolate’, and then I thought, ‘No, that was the old way of doing things, you don’t get up and think you're going to have a bar of chocolate, go and have a piece of fruit’ (Patient 1015)

These skills included self-monitoring (eg, discussing a physical activity log with the group); relapse prevention (eg, making a plan for getting back on track); social network mapping; noticing emotional or negative self-talk, examining thinking patterns and brainstorming alternatives; and managing portion control:

I said, ‘well, I can’t have that’ […] but she [facilitator] said ‘why can’t you eat it?’ and she’s like ‘nothing’s off limits. It’s just about your portion control.’ It was almost a bit of a light-bulb moment (Patient 2031).

Another patient learnt that

if you have a bad day it doesn’t have to be a bad week and if you’d, you start again, it’s not a failure (Patient 3020).

An outcome for patients was they did share problem solving and goal-setting, discuss weight management ‘solutions’ (eg, brainstorming solutions to emotional eating) and what goals were achievable:

just making small changes you know, not doing everything at once and reinforcing you know, “This is not a diet, it’s a lifestyle change,” and, “It’s not a quick fix, it’s not going to happen overnight” (Staff 04).

One way facilitators reinforced patients’ capability was by drawing into group discussions and activity the quieter members who could nevertheless contribute practical ideas:

she’s going ’so I’ve been planning, changing what I eat, I’m looking at labels, I’m doing the exercises’…I wonder if she’d been more vocal took part in more of the earlier group whether that that might have spurred other people on to say 'oh you tried that, and that’s worked, I might give that a go’ (Facilitator 02).

Negative comments concerned the session content:

the information they gave was a little bit school level, if that makes sense? Most people should already know these things… it could have been focusing on each individual person and why they feel they’re there (Patient 1005).

As a context, patients’ trust in the facilitators’ competence also seemed to increase their own confidence:

you think to yourself [… names] are trained in their profession and they know what they’re talking about. There’s more clarification in what they’re saying and a bit more clout about it than what I was getting out of Slimming World. (Patient 2031).

#### Group processes strengthen motivation (mechanism 7)

An apparent outcome of the emergent group processes for the patients was to establish social connections and a social identity which helped reinforce their motivation for behaviour change. Patients already versed in weight management methods valued PROGROUP more for its motivational and group-process content. This included greater self-awareness:

Obviously, talking about your habits and things like that is always good to talk about, because you might recognise something that you’re not realising you do, but […] Most people should already know these things… it [PROGROUP] could have been focusing on each individual person and why they feel they’re there (Patient 1005).

Facilitators encouraged group members to report successes (reinforcing motivation) and how they did so (reinforcing capability):

the lady who set the group up she’d be like, ‘right, come on, list your wins on the group!’, so it was just about keeping it in the mindset […] And sometimes people just put ‘who’s having a good week?’ and we share funny memes, and I’ve found it really nice (Patient 2031).

Such skills were therefore an important context for Mechanism 7. As another, the facilitators (and the logic model) initially assumed that it helped to have a relatable ‘buddy’ within the group as a behavioural model, but for the patients themselves it could also be someone outside, even fictional, provided the patient could identify with them:

there was one [vignette] about Louise—she dropped her kids off and then… because it was familiar to me I think, because I have two children, I’m a busy mom (Patient 2052).

Further outcomes for patients included maintaining motivation despite what might otherwise have seemed a failure.

I’ll go a month to six weeks, everything be fine, but then […] I’ll have a few beers and maybe a curry and all this other stuff […] The following morning, I thought ‘Ah God there you go, you’ve blown it now’, and then I said ‘No you haven’t you’ve just had one day out of how many hundreds’ […] Put a few pounds back on but then just get back on it and start again (Patient 3009).

Feelings of relief and confidence (again) increased patients’ willingness to try further behaviour change techniques. For patients, the group dynamics become in this respect self-reinforcing.

#### Increased capability and motivation support use of behaviour change methods at home (mechanisms 8–9)

Group members’ enhanced capability and motivation, and the maintenance phase of PROGROUP, were intended jointly to stimulate ongoing behaviour change at home, an outcome which for at least some patients did occur:

I have become more aware of what I am eating and as to whether I am really hungry or thirsty (Feedback questionnaire).

Others described learning to alter patterns such as eating between meals or learning to snack on fruit instead of chocolate. Successful weight control could then become self-reinforcing:

when I went for my six month weighing, I think I weighed virtually what I weighed the day I went to [site] the first time, which for me was a result, because I didn't weigh any more. […] when I go for my twelve month weigh in, I will be getting my PROGROUP book out more and I will weigh less (Patient 1015).

This affected other activities too, for instance

to go out and meet more people […] reconnect with a few friends, we’ve been out for a few coffees and talked about a few things, not just weight but different things and just catch up really (Patient 2052).

These patients attributed these to the psychological tools (i.e. capability) learnt through PROGROUP:

What PG has done is establish those routines […] What I do now is choose a day in the week and I sit down on a Sunday with my kids and make a list of foods that I have in the house or fridge and make a food plan for the week (Patient 2052).

Group support contributed

if there’s something bothering them or they haven't had a good week or whatever it is, then that that was the ideal platform for them to vent their problems. We were the right crowd to listen, because we understood (Patient 3009).

As a context, support from others at home facilitated these changes. One patient (2052) described using a fitness app together with her daughter, another that

I would speak to my husband about what we did, how I felt it went and things like that, but I haven’t necessarily got the book out and spoken through it (Patient 1005).

and a third how

my husband he’s never been brilliant at cooking but he tries now, he does most of it, he knows what he’s cooking… because obviously then at least it keeps me going and I can see what goes in everything (Patient 3020).

In these ways PROGROUP appeared to shape patient attitudes and behaviour outside the treatment setting, even have an ‘intervention contagion’ by affecting the actions of those who were connected to the patient but weren’t themselves group members.

#### Preparation for long-term behavioural maintenance supports use of behaviour change methods at home (mechanism 10)

The latter phase of the intervention was to prepare the group members to continue their behaviour change outside the treatment setting when formal treatment ended. Some patients were sad at the prospect of group sessions ending but hoped to still meet afterwards.

The intervention encouraged patients to create a WhatsApp group to help support each other outside the group sessions. Three out of the four groups set up online groups through which they shared advice and recipes, and mutual support. That helped in

Just making everyone gel. Even as silly as, once we’d actually got it up and running we were sharing pictures of our cats and dogs. […] .It gave us something to chat about when we got there at the beginning of the session (Patient 2054).

For instance,

people that have their bad days, we give them advice and stuff, and we check in to let each other know what we’ve been doing (Patient 2015).

Patients also held non-virtual group activities outside the formal sessions:

I know some of them were meeting up last weekend, so yeah I feel like there was a friendship element to [group] (Facilitator 05).

In these ways, the group was internalised and sustained. Patients saw it as more than just a venue for receiving intervention.

As the groups became more self-sustaining for patients, the role for facilitators reduced to

trying to give them more time to hear their own voices, me taking that backward seat, and allow[ing] more interaction to happen so for me I’d be more conscious [… that what] I need to do is do nothing and say nothing and allow that question to you know be addressed and discussed in themselves, among themselves (Facilitator 03).

Again, having sufficient time to establish and consolidate these connections was a context affecting how effective the group-initiated networks were. Some facilitators encouraged these groups, but others needed further training about what the on-line group was for and how to use it (eg, as a group resource for conversation, mutual support and problem-solving).

The outcomes for patient behaviour change at home were to reinforce mechanisms 8–9, as described above.

### Logic model revision

**Figure 2:**
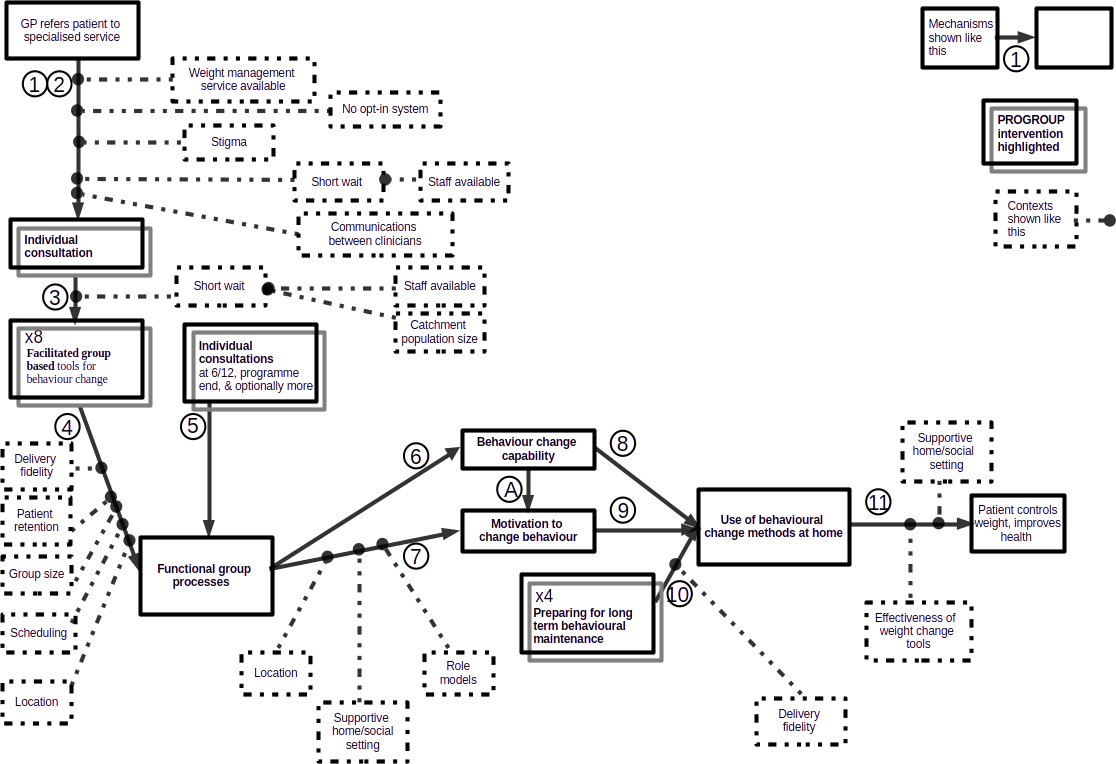
PROGROUP revised logic model shows the whole revised logic model diagrammatically. For comparability, it keeps the same mechanism numbers as figure 1, despite collapsing the first two mechanisms and adding mechanism A. GP, general practitioner.

These findings suggested revisions to the initial logic model. The two-stage referral system (mechanisms 1 and 2) sometimes worked slowly and erratically. A second (and in one site, a third) referral stage gave opportunity for delay and miscommunication. The provision of a weight management intervention by GP networks suggests collapsing mechanisms 1 and 2 into one, with the PROGROUP facilitator alone screening patients for suitability. For PROGROUP purposes, it was not necessary to record patients’ weight at this stage. Our findings suggest (see mechanism 3) narrower patient selection criteria for PROGROUP and the presence of an additional mechanism which we had not initially anticipated: the development of personal capability (efficacy) reinforces the motivation for behaviour change (mechanism A in figure 2). This additional mechanism was not an added interventional activity but an unforeseen positive outcome for patients of the group sessions (see mechanism 6). Our findings also suggested revising the facilitator training (detailed report being prepared for publication).

Revisions to the assumed contexts were more numerous. For mechanisms 1–4, support outside the treatment setting and stigma attaching to obesity were less important contexts than the initial logic model assumed. Patient retention was more important for mechanism 4 than earlier. Although mundane, organisational contexts such as timetabling, duration, location, pressure on accommodation and staff were practically important contexts influencing patient retention.

Table 2 summarises these changes, marking with asterisks the necessary revisions to the initial logic model.

**Table 2:** The revised logic model

Mechanisms	Facilitating context(s)
GP refers patient	GPs aware of services, have interest, time to discuss, non-stigmatising attitude Specialist weight management service provider available*
Specialist service refers to PROGROUP	Suitable screener(s) available Sufficient patients Reliable communication within weight management services Short waiting time, depends on staff availability* Patient opt-out rather than opt-in referral system*
From individual consultation to patient group: Narrower patient selection criteria*	Group size Location for group accessible within acceptable journey time, hence distance, for physically disabled people* Scheduling practicable for patients* Short waiting time, depends on size of patient catchment population*
Individual consultations contribute to functional group processes.	Fidelity of facilitators’ programme delivery
4,6,7. Group sessions produce functional group processes, hence capability and motivation Group motivation and dynamics content* Role models or ‘buddy’ can be external or fictional*	Fidelity of facilitators’ programme delivery, including group dynamics content* Sufficiently large, stable group, hence patient retention* Location of group sessions* Scheduling of group sessions* Patient trust in facilitators’ competence*
8,9 Motivation and capability increase use of behaviour change methods at home	Support outside the treatment setting
Preparation for behavioural maintenance increases use of behaviour change methods at home	Facilitators have necessary skills* Sufficient time for group members to replace the facilitator’s input
AIncreased capability reinforces motivation*	(None)

* indicates change from initial model

GP, general practitioner.

The findings also suggest what variations in its delivery components that PROGROUP could tolerate without compromising the effectiveness of the programme components (table 3).

**Table 3:** Delivery components: variations compatible with effective programme components

Mechanisms	Conditions necessary for delivery	Observed variants (respectively); *further possibilities in italics*
GP referral to specialist weight management service	Specialist weight management service provider available GPs who are aware of services available, have interest, time to discuss and a non-stigmatising attitude	CHS, GP network, *third sector provider, polyclinic, mental health service* *Practice nurses, advanced nurse practitioners replace GP in chronic disease management*
Specialist weight management service referral to PROGROUP	Suitable screener(s) available Sufficient patients BMI recorded	Public health practitioner, physiotherapist, clinical psychologist, dietician, *psychiatrist, physician, nurse practitioner* Referral rate ≥12 per month. BMI recorded by GP, initial screener, first individual consultation or first group session; *or self-report*
From individual consultation to patient group.	Group size Location for group accessible within acceptable journey time, hence distance, for physically disabled people Scheduling practicable for patients One-to-one consultation	Range 15–18, *15–30*. Distance <60 km Weekday or week-end Face-to-face, by telephone or video conference
4,5,6. Group sessions produce group processes, hence capability and motivation.	Facilitator training completed Sufficiently large, stable group Location	Facilitators trained face-to-face and/or online Group attendance of at least five, hence initial group size large enough to maintain this despite drop-outs Hospital or accessible local community venue, distance <60 km
7,8 Motivation and capability produce behaviour change at home.	Implemented by patients	
Group maintenance activities reinforce mutual support and individual weight management activities.	As mechanism 4	As mechanism 4, but 4 sessions
Individual consultations contribute to behaviour change at home.	As mechanism 3	Individual vs batch scheduling Face-to-face vs on-line

BMI, body mass index; CHS, community health service; GP, general practitioner.

### Strengths and limitations

We found the initial logic model assumptions about the PROGROUP mechanisms were largely valid, but there was scope to simplify and qualify some, and add a previously unrecognised reinforcement mechanism that the intervention also established. The assumptions about what contexts affected the PROGROUP mechanisms required various additions (eg, recognising the impacts of group size, stability, location and scheduling). The intervention appeared to produce outcomes of the kinds intended.

Other studies have found some similar patterns to those reported above. Fragmented referral routes and systems and poor communication between weight management services have been reported for other weight management services.^44 45^ So has attrition of patient groups, indeed higher attrition than in PROGROUP to date,^48 49^ and that patients’ prior work, education or care commitments limited their scope to access services,^50^ as did transport difficulties.^51 52^ Staff and service overload was a barrier to implementing PROGROUP, further evidence that a degree of ‘organisational slack’ is necessary for innovation.^53^ Earlier studies also found that stable membership facilitated group formation and work,^45^ and that patients tended to value social interaction in itself.^54^

Because the implementation of PROGROUP only began 2 years ago, the above findings do not cover any aspects of PROGROUP implementation that might take longer to emerge, its economic consequences, nor how PROGROUP compares with other behavioural weight management programmes. This paper reports qualitative findings about what kinds of mechanisms PROGROUP established, triggered and used, what kinds of outcomes they produced, and what kinds of context affected those outcomes. As noted, an RCT will quantify the effects on weight change and other health outcomes including psychological well-being. Since some patients dropped out from the intervention, those retained were likely to be the most supportive.

Besides its empirical findings, this study adds to the literature a qualitative proof-of-concept of the PROGROUP intervention. It shows how a realist analysis of a pre-RCT feasibility study can be used to make the intervention’s logic model more securely evidence-based. It offers an initial explanation of the relationships between the programme and delivery components of an intervention. In group-based interventions such as PROGROUP, the programme and delivery components are conceptually but not always practically distinct. A mechanism can be both at once. In PROGROUP, such were the initial consultations and the group maintenance meetings, among other components. Furthermore, the programme and delivery components have to fit each other. The size, number, frequency, duration and location of PROGROUP meetings (delivery components) constrained the extent to which group identity and collaborative action could develop (programme components). Therefore, the delivery components (eg, sufficient adequately trained staff, accessible venues) had to be adapted to what the programme components required. The delivery components can often be implemented in alternate, equally effective ways (eg, different referral routes, facilitators from different professions), creating a well-defined latitude in what delivery components are practicable (table 3). The programme components thus constrain, rather than determine, what delivery components are required. While it might make practical sense to optimise (specify ‘the one best’)^55^ programme component design, that does not necessarily apply to delivery components.

By exposing the mechanisms through which an intervention works, a realist analysis also suggests whether that logic model appears extendible to other care groups. The social identity approach to health provides a framework to inform the development of group-based behavioural interventions.^56^ Because nothing in its design was unique to severe obesity, the above logic model appears generally applicable to conditions with the characteristics of:

1. being susceptible to mitigation, even reversal, during the patient’s daily life outside treatment settings, by means of…
2. the patient habitually applying a certain repertoire of behaviours, which requires…
3. the patient developing stable, long-term motivation, attention and behaviour change, which…
4. support from fellow patients helps sustain.

Such interventions may include aspects of rehabilitation after injury or illness, or for some neurological conditions, but to test that will require further research.

## Conclusions

This paper reports a method for empirically testing and developing the logic model of an intervention before full-scale clinical trial or widespread adoption. In qualitative terms of what kinds of mechanisms could be established and used, what kinds of outcomes they produced, and in what contexts, the above findings largely corroborated PROGROUP’s initial logic model. Nevertheless, some adjustments were also necessary. One concerned delivery components (possible simplification of referral routes). Others (narrower patient selection criteria, greater emphasis on group dynamics) concerned programme components. The findings also suggested more extensive revisions of the assumptions about what contexts, including some delivery components, facilitated or impeded the mechanisms that PROGROUP used. Lastly, an intervention cannot necessarily influence all the contexts which affect what outcomes it produces: in PROGROUP, support from the patient’s family, and overall health system capacity and overload. Such contexts are neither delivery nor programme components of the intervention, although they may critically influence its outcomes.

## Data Availability

Data are available on reasonable request.
