# Supplementary material for: Logic model development through a feasibility RCT for a group-based weight management programme
Source: BMJ Open. 2026 Mar 24;16(3):e094569. doi: 10.1136/bmjopen-2024-094569 (PMC13034288; doi:10.1136/bmjopen-2024-094569)
Supplement: Supplementary file 1 [file bmjopen-2024-094569.reviewer_comments.pdf]

PEER REVIEW HISTORY

BMJ Open publishes all reviews undertaken for accepted manuscripts. Reviewers are asked to complete a checklist review form and are provided with free text boxes to elaborate on their assessment. These free text comments are reproduced below.

ARTICLE DETAILS

Title (Provisional)

Logic model development through a feasibility RCT for a group-based weight management programme

Authors

Sheaff, Rod; Moghadam, Shokraneh; Hollands, Laura; Hawkins, Lily; Swancutt, Dawn; Lloyd, Jenny; Pinkney, Jonathan; Tarrant, Mark

---

VERSION 1 - REVIEW

**Reviewer**	**1**
**Name**	**Nguyen, Dieu**
**Affiliation**	**Deakin University, Deakin Health Economics**
**Date**	**19-Mar-2025**
**COI**	**None**

Thank you for a beautiful manuscript!

I have only had some minor suggestion as below:

1. In background information, it might be better to have a high level summary of "PROGROUP" instead of long text, with a clear rationale, aim, objective (which the authors had)

2. Lines 163-219 seem to belong to method

3. What is realist evaluation? could the author define it clearly so people who are not familiar with this field have a better idea of what it refers too?

4. In finding, some reported results might need a minor revision to ensure it capture the realist evaluation theory cycle (Pawson and Tilley, 1997): what works, for whom, under what circumstances, in what context and/or mechanism, and what is the outcome. For example Mechanism 5: Individual consultations contribute to functional group processes- could the authors provide some supportive quote(s)/evidence?

4. Some minor referencing error

**Reviewer**	**2**
**Name**	**graham, Henrietta**
**Affiliation**	**Loughborough University**
**Date**	**23-Jul-2025**
**COI**	**None**

Overall general comments

Testing the logic model prior to conducting a larger feasibility trial is a valuable and commendable approach. Proof-of-concept studies such as this are often underutilised, yet they have the potential to significantly inform the design of effective weight management interventions. However, the clarity of the manuscript, particularly in the introduction and methods section, is very poor, which unfortunately limits the overall usefulness and impact of the paper.

Abstract

In general, I think it is very difficult to know what this study is about from just reading the abstract – it is very confusing – I have added further notes below.

The objectives are currently phrased in an unclear and ambiguous manner. Providing clearer articulation and separating them into distinct, well-defined components would enhance their clarity and improve the overall usefulness of the manuscript.

Could the outcomes be listed closer to the objectives section? It would be easier to understand the objectives knowing the outcomes sooner.

The primary and secondary outcomes measures are a bit vague and unclear – could you explain these a bit more?

It is unclear how the stated objectives are aligned with the specified outcomes. The manuscript states that the objective is to assess whether PROGROUP produces the intended outcomes; however, these intended outcomes are not clearly defined.

Furthermore, the primary outcome is described as identifying the mechanisms that PROGROUP establishes and triggers, but it is not clear how this is being measured. Greater clarity and further explanation of the linkage between objectives, outcomes, and measurement methods would significantly improve the coherence and interpretability of the study.

It isn't clear which outcomes are the primary or secondary ones – could you please clarify.

In the results section – when you say "the logic model assumed…" is this the revised logic model or the original logic model?

In the results section – you don't include what kinds of behaviours resulted from the intervention – is behaviour change outside the treatment setting one of them? You haven't made this clear.

Introduction

The introduction is quite disjointed and does not flow – e.g., on lines 104 you provide general background on obesity, then move on to talk about something else then on lines 130 you go back to giving general background on obesity.

Could you please use more up-to-date citations in the introduction please? Some of these are over 22 years old and there is more up to date evidence available in some circumstances.

Line 108: consider rephrasing the term "low levels of obesity" – do you mean category I obesity?

Line 112: "which appears extensible for use with other care groups" – what care groups? Please specify. Do you have any evidence to support this? Citations?

Line 115: "Implementing it puts these assumptions to a practical, empirical test". Implementing the logic model or the intervention? Please specify.

Line 145: Consider rephrasing how you have written this – "simple obesity" may not be appropriate.

I do not understand how the research described on lines 155-159 relates to the question raised on lines 154 and 155.

Figure 1 is quite poor quality, so it is hard to read – although I understand this is not the fault of the authors.

Sentence from line 210 – 214 is very long – consider rephrasing to concisely convey your point.

Methods

The methods section is better written than the abstract and introduction but still some issues, which I have described below.

You say that you addressed the three questions in three stages. Not clear what information relates to stage 3?

Did the feasibility trial protocol that you published include the logic model?

Were the researchers who analysed the data completely independent? What was their relationship to the people who designed the intervention?

Good to see involvement of PAG, Steering Committee and PPI in the study.

Results

Some interesting findings in here – e.g., information about unforeseen selection questions could be useful to others designing similar interventions.

I think the details that are provided about what revisions are needed to the logic model are well written – useful to know which mechanisms were assumed too important and not important enough etc.

Again, Figure 2 is difficult to see but I know this is not the fault of the author.

Line 435: Would it not have been better to further explore this further with the participant instead of inferring?

Interesting that none of the quotes included negative feedback about the intervention.

Did you receive any negative feedback?

Discussion

The overall summary of findings in the discussion is accurate based on what was written in the results and findings are linked to other literature.

Acknowledged some of the limitations of the study, which was good.

VERSION 1 - AUTHOR RESPONSE

Reviewer suggestions for amendments	Author response
Reviewer: 1	
1. In background information it might be better to have a high level summary of "PROGROUP" instead of long text with a clear rationale aim objective (which the authors had)	Lines 163-219 describe the PROGROUP intervention logic model in detail, which was the first stage of the method reported in the Methods section. Therefore we have addressed both these points by treating lines 182-219 as preliminary findings, and now repositioned them immediately after the Methods section and before the other findings.
2. Lines 163-219 seem to belong to method	
3. What is realist evaluation? could the author define it clearly so people who are not familiar with this field have a better idea of what it refers too?	Text added at start of Methods section, with reference linked.
4. In finding some reported results might need a minor revision to ensure it capture the realist evaluation theory cycle (Pawson and Tilley 1997): what works for whom under what circumstances in what context and/or mechanism and what is the outcome. For example Mechanism 5: Individual	We have now explicitly stated the context, outcome and beneficiaries for each mechanism in the initial logic model. Material added on Mechanism 5.

Commented [RS1]: Shok, this refers to the second and/or third individual consultation. Have we any direct evidence of patients' responses to them please?

consultations contribute to functional group processes- could the authors provide some supportive quote(s)/evidence?	
4. Some minor referencing error	Checked and corrected
Reviewer: 2	
Abstract	
The objectives are currently phrased in an unclear and ambiguous manner. Providing clearer articulation and separating them into distinct well-defined components would enhance their clarity and improve the overall usefulness of the manuscript.	Re-drafted.
Could the outcomes be listed closer to the objectives section? It would be easier to understand the objectives knowing the outcomes sooner.	The abstract headings and their sequence follow the journal's submission guidelines at <https://bmjopen.bmj.com/pages/authors#submission_guidelines> which we hesitate to depart from unless the editor(s) so advise.
It is unclear how the stated objectives are aligned with the specified outcomes. The manuscript states that the objective is to assess whether PROGROUP produces the intended outcomes; however these intended outcomes are not clearly defined.	Alignments now stated in the abstract, and outcomes more clearly formulated.
The primary and secondary outcomes measures are a bit vague and unclear – could you explain these a bit more? [...] Furthermore the primary outcome is described as identifying the mechanisms that PROGROUP establishes and triggers but it is not clear how this is being measured. Greater clarity and further explanation of the linkage between objectives outcomes and measurement methods would significantly improve the coherence and interpretability of the study.	We may be at crossed purposes with Reviewer 2 here. As what are now lines 55-59 and 184-7 explain, the present qualitative analysis is not designed to quantify (measure) the relationships between mechanism, so neither measurements nor measurement methods are necessarily germane to this stage of logic model development. (They are of course relevant to the RCT for which the present study prepared.) Rather, the present aim is is to establish qualitatively what causal connection(s) obtain between them. We have amended to text to make this clearer.
It isn't clear which outcomes are the primary or secondary ones – could you please clarify.	Because this study is not a clinical trial but uses qualitative methods, the outcomes are not quantified (measured). Distinguishing the primary and secondary outcomes is indeed necessary for a statistical analysis of effect size, but that is not what this paper concerns, as lines 55-59 and 184-7 explain. Nevertheless, we now distinguish the two.

In the results section – when you say "the logic model assumed…" is this the revised logic model or the original logic model?	Adjective added.
In the results section – you don't include what kinds of behaviours resulted from the intervention – is behaviour change outside the treatment setting one of them? You haven't made this clear.	We have added that they include behaviours outside the treatment setting.
Introduction	
The introduction is quite disjointed and does not flow – e.g. on lines 104 you provide general background on obesity.	Text re-edited and text re-sequenced as noted in response to R1, to improve flow and remove duplication.
Could you please use more up-to-date citations in the introduction please? Some of these are over 22 years old and there is more up to date evidence available in some circumstances.	Updated.
Line 108: consider rephrasing the term "low levels of obesity" – do you mean category I obesity?	Yes, Text changed accordingly.
Line 112: "which appears extensible for use with other care groups" – what care groups? Please specify. Do you have any evidence to support this? Citations?	Reworded as question. Later, the paper notes the need for further research to discover such evidence. Since it doesn't yet exist, we can't cite it; but we do define the four conditions stating how far the intervention might be *prima facie* extensible.
Line 115: "Implementing it puts these assumptions to a practical empirical test". Implementing the logic model or the intervention? Please specify.	Text revised accordingly.
Line 145: Consider rephrasing how you have written this – "simple obesity" may not be appropriate.	Redrafted
I do not understand how the research described on lines 155-159 relates to the question raised on lines 154 and 155.	Text moved up, where it fits better.
Figure 1 is quite poor quality so it is hard to read – although I understand this is not the fault of the authors.	This might be dealt with at editing/proofing stages should the revised paper be accepted.
Sentence from line 210 – 214 is very long – consider rephrasing to concisely convey your point.	Sentence divided.

Commented [RS2]: Jon – is this the right answer?

Methods	
You say that you addressed the three questions in three stages. Not clear what information relates to stage 3?	Cross-reference added.
Did the feasibility trial protocol that you published include the logic model?	It mentions the logic model but does not describe it in detail, so we have added that the original grant application did describe the programme components.
Were the researchers who analysed the data completely independent? What was their relationship to the people who designed the intervention?	Note added.
Results	
Again Figure 2 is difficult to see but I know this is not the fault of the author.	This too might be dealt with at editing/proofing stages should the revised paper be accepted.
Line 435: Would it not have been better to further explore this further with the participant instead of inferring?	In fact, we did. Text amended to make that clearer.
Interesting that none of the quotes included negative feedback about the intervention.<br>Did you receive any negative feedback?	We now report the negative reaction of a patient who was awaiting bariatric surgery. Considering that there were drop-outs from the intervention, the retained patients were likely to be the most supportive. This is now noted as a study limitation.

Commented [RS3]: Shok – were there any others?
